# Towards the neuromorphic Cyber-Twin: an architecture for cognitive defense in digital twin ecosystems

**DOI:** 10.3389/fdata.2025.1659757

**Published:** 2025-11-04

**Authors:** Nida Nasir, Hussam Al Hamadi

**Affiliations:** ^1^COSYS-LEOST Laboratory, Universit Gustave Eiffel, Lille, France; ^2^College of Engineering and IT, University of Dubai, Dubai, United Arab Emirates

**Keywords:** neuromorphic computing, digital twins, spiking neural networks, cybersecurity, cognitive defense, adaptive learning, STDP, event-driven processing

## Abstract

**Introduction:**

As cyber-physical systems become increasingly virtualized, digital twins have emerged as essential components for real-time monitoring, simulation, and control. However, their growing complexity and exposure to dynamic network environments make them vulnerable to sophisticated cyber threats. Traditional rule-based and machine-learning-based security models often fail to adapt in real time to evolving attack patterns, particularly in decentralized and resource-constrained settings.

**Methods:**

This study introduces the Neuromorphic Cyber-Twin (NCT), a brain-inspired architectural framework that integrates spiking neural networks (SNNs) and event-driven cognition to enhance adaptive cyber defense. The NCT leverages neuromorphic principles such as sparse coding, temporal encoding, and spike-timing-dependent plasticity (STDP) to transform telemetry data from the digital-twin layer into spike-based sensory inputs. A layered cognitive architecture continuously monitors behavioral deviations, infers anomalies, and autonomously adapts its defensive responses in alignment with system dynamics.

**Results:**

Lightweight prototype simulations demonstrate the feasibility of NCT-based event-driven anomaly detection and adaptive defense. The results highlight advantages in low-latency detection, contextual awareness, and energy efficiency compared with conventional machine-learning models.

**Discussion:**

The NCT framework represents a biologically inspired paradigm for scalable, self-evolving cybersecurity in virtualized ecosystems. Potential applications include infrastructure monitoring, autonomous transportation, and industrial control systems. Comprehensive benchmarking and large-scale validation are identified as future research directions.

## 1 Introduction

The digital transformation of physical infrastructure has ushered in a new era of intelligent systems, with Digital Twins (DTs) becoming central to the modeling, monitoring, and control of cyber-physical environments (Kreuzer et al., 2024). These high-fidelity virtual replicas facilitate real-time synchronization between physical assets and their digital counterparts, fostering applications in smart cities (Peldon et al., 2024), industrial automation, transportation, and healthcare. However, as DTs become more autonomous and interconnected, they also become high-value targets for cyber threats, ranging from data manipulation and spoofing to advanced persistent attacks that evolve over time (Hassija et al., 2025).

Traditional cybersecurity mechanisms, including rule-based intrusion detection systems and supervised machine learning classifiers, offer limited adaptability and often struggle with high false positives, insufficient context awareness, and energy inefficiency (Mohamed, 2025). These limitations are especially problematic in virtualized ecosystems, where real-time responsiveness and low computational overhead are critical. Moreover, current approaches typically lack the cognitive flexibility to anticipate emerging threats or respond meaningfully in dynamic, distributed environments.

Inspired by the adaptive intelligence of biological systems, neuromorphic computing presents a promising new avenue for designing cyber defense architectures that are both energy-efficient and context-aware. Specifically, SNNs offer event-driven computation and local learning mechanisms such as STDP, making them well suited for modeling dynamic behavioral patterns and identifying anomalies without relying on labeled data or centralized processing (Farsa et al., 2025).

In this work, we present a conceptual analysis of the Neuromorphic Cyber-Twin, a novel architectural framework that integrates neuromorphic intelligence directly into the Digital Twin layer to enable autonomous, cognitive cybersecurity. Unlike conventional DTs that passively mirror physical states, the NCT actively senses, interprets, and reacts to threats in a manner inspired by the brains adaptive immune and learning systems. As a conceptual contribution, the emphasis is on theoretical grounding, architectural vision, and prototype feasibility, while comprehensive empirical benchmarking is identified as a direction for future research.

The contributions of this paper are threefold:

- We propose a layered architecture for the NCT that incorporates spike-based sensing, temporal encoding, neuromorphic inference, and adaptive policy execution.
- We present the theoretical foundations behind its cognitive processing model, drawing from biological learning principles and computational neuroscience.
- We discuss potential use cases across virtualized infrastructure domains and outline key research challenges for realizing neuromorphic cyber defense in practice.

This work lays the groundwork for a new class of intelligent, self-defending digital systems capable of learning and evolving alongside their cyber-physical counterparts.

## 2 Background and related work

Recent studies demonstrate the growing maturity of neuromorphic and Digital Twin security research. For example, Roy et al. (2019) and Schuman et al. (2022) highlight spike-based models as a foundation for energy-efficient and context-aware inference, while Yan et al. (2019) demonstrate large-scale distributed neuromorphic hardware suitable for real-time anomaly detection. More recent work has explored hybrid DT-AI frameworks for cyberphysical security and neuromorphic computing integration in safety-critical IoT deployments, reinforcing the timeliness of the NCT approach. Building on these advances, this article contributes to a unified architectural vision that emphasizes spiking-based cognitive defense, scalable federation, and reproducibility (Uludağ et al., 2024; Lutes et al., 2025; Muir and Sheik, 2025). In this section, we first examine the cybersecurity needs of DTs, followed by an exploration of related efforts in neuromorphic computing and intelligent anomaly detection.

### 2.1 Digital twins and cybersecurity needs

DTs serve as virtual real-time representations of physical systems, enabling predictive maintenance, operational optimization, and autonomous control. As they become central to the functioning of critical infrastructure, such as smart grids, transportation networks, and industrial control systems, their exposure to cyber threats increases significantly. Malicious actors can manipulate DT data streams, inject false telemetry, or interfere with their synchronization logic to cause physical disruptions or misinformed decisions (Holmes et al., 2021).

Despite their importance, current DT implementations often rely on conventional IT-centric security models that are reactive, centralized, or computationally heavy. These models do not account for the continuous, high-dimensional, and event-driven nature of DT environments, nor do they provide mechanisms for adaptive learning or contextual defense (Homaei et al., 2024).

### 2.2 Neuromorphic computing and spiking neural networks

Neuromorphic computing is an emerging paradigm inspired by the structure and function of the human brain. At its core are SNNs, which process information through discrete time-encoded events (spikes), offering advantages in temporal sensitivity, energy efficiency, and local learning. Unlike traditional neural networks, SNNs do not require dense input vectors or continuous processing; they operate on sparse, asynchronous data and support biologically plausible mechanisms such as STDP for unsupervised adaptation (Auge et al., 2021). These properties make SNNs ideal candidates for real-time anomaly detection, context-sensitive decision-making, and energy-aware computation, key requirements for intelligent cyber-defense systems embedded in DT environments (Bäßler et al., 2022).

### 2.3 Cybersecurity in neuromorphic and DT contexts

Recent works have investigated neuromorphic systems in various applications, including signal classification, IoT anomaly detection, and physical-layer intrusion detection. However, these efforts are primarily focused on low-level signal processing or specific application domains and do not propose an architectural integration of SNN-based cognitive reasoning directly within the Digital Twin layer. The cognitive modeling and autonomous reaction capabilities required for high-level defense in virtualized systems remain unexplored (Korki et al., 2022).

### 2.4 Positioning of this work

In this paper, we bridge this gap by proposing the NCT: a layered cognitive security model for Digital Twins, empowered by neuromorphic learning and real-time adaptation. Unlike conventional anomaly detection pipelines, the NCT continuously monitors the DT's behavior, identifies cognitive anomalies, and adapts policy decisions using SNN-driven internal reasoning loops.

### 2.5 Literature landscape and positioning

#### 2.5.1 Neuromorphic cybersecurity architectures

Recent efforts in neuromorphic cybersecurity have explored spiking-based intrusion detection, radio signal classification, and low-power threat inference. For instance, Loihi-based platforms have demonstrated on-chip learning for detecting anomalies in network traffic using spike-timing-dependent plasticity. Similarly, BrainChips Akida SoC has been applied in edge AI use cases for environmental and behavioral anomaly detection. However, these works primarily operate at the physical or signal level and do not engage with the abstraction or autonomy layer introduced by Digital Twins (Zahm et al., 2022).

More recent studies further advance this direction. For example, Farsa et al. (2025) survey FPGA-based neuromorphic implementations that target security-critical workloads, demonstrating reconfigurable hardware support for adaptive defense. Xu et al. (2024) explore federated reinforcement learning in V2X networks, providing distributed intelligence that aligns closely with our federated neuromorphic vision. At the algorithmic level, Nazari and Amiri (2025) propose a biologically inspired fast STDP learning approach, while Stadtmann et al. (2023) investigates robust local learning on neuromorphic hardware. Collectively, these works reinforce the timeliness of embedding spiking-based cognition into cybersecurity systems and highlight the gap that the NCT addresses by extending beyond low-level anomaly detection to cognitive, digital-twin-aware defense.

#### 2.5.2 Hybrid DT–AI security frameworks

Digital Twins have increasingly integrated AI models for predictive maintenance, fault diagnosis, and anomaly detection. Hybrid architectures often involve machine learning algorithms that analyze DT telemetry to detect abnormal behaviors or recommend control actions (Alfaro-Viquez et al., 2025). Nevertheless, such systems are typically trained offline, require high-quality labeled datasets, and lack mechanisms for real-time adaptation under data drift or adversarial manipulation.

#### 2.5.3 Contribution of this work

To the best of our knowledge, this paper presents the first architectural and theoretical formulation of a *neuromorphic cognitive layer embedded within a Digital Twin framework*. The proposed NCT advances the state-of-the-art by:

- Embedding online, unsupervised SNNs for cyber defense within the DT layer,
- Offering a biologically inspired feedback loop for local inference and adaptation,
- Proposing a federated expansion of neuromorphic DTs for secure, scalable collaboration.

This uniquely positions NCT between traditional ML-enhanced DTs and edge-deployed neuromorphic security primitives, bridging the gap between abstraction, autonomy, and adaptive defense.

## 3 Conceptual framework: the neuromorphic Cyber-Twin

Building on the gaps identified in conventional DT security solutions, this section introduces a neuromorphic alternative inspired by cognitive neuroscience and event-driven processing. The proposed NCT aims to embed adaptive intelligence directly into the DT architecture, enabling it to autonomously perceive, infer, and respond to anomalies in real time.

### 3.1 Overview and motivation

The *NCT* is conceived as a next-generation cybersecurity architecture that combines biologically inspired intelligence with virtualized Digital Twin environments. Unlike conventional Digital Twins that passively mirror physical states, the NCT introduces an active, cognitive layer capable of recognizing, learning from, and responding to security threats in real time. The proposed framework embeds a neuromorphic processing unit, primarily a SNN, within the DT control loop to form an intelligent anomaly detection and response module.

### 3.2 Functional design objectives

The design of the NCT is guided by four primary objectives:

1. Real-time threat monitoring: Continuous analysis of DT telemetry for deviations or anomalies.
2. Low-power cognitive reasoning: Event-driven SNN-based processing for energy-efficient inference.
3. Adaptive behavior: Online learning through STDP to respond to emerging threats without human intervention.
4. Autonomous actuation: Enabling local response policies within the DT ecosystem based on anomaly context.

### 3.3 Layered architecture of the NCT

The NCT architecture is structured into five core layers:

- Physical-Digital Interface Layer: Gathers real-time data from the physical system (e.g., sensor telemetry, actuator feedback) and maps it to the DT model.
- Spike Encoder Layer: Converts multivariate DT state features into spike trains using biologically inspired methods such as rate coding, latency coding, or time-to-first-spike encoding (Auge et al., 2021).
- Neuromorphic Inference Core: A multi-layer SNN trained with unsupervised learning rules (e.g., STDP) that identifies anomalies based on temporal spike pattern deviations (Bäßler et al., 2022).
- Decision and Actuation Layer: Interprets spiking activity using winner-take-all or voting mechanisms to initiate security actions such as data isolation, subsystem rollback, or alert generation.
- Feedback and Memory Loop: Supports lifelong learning by integrating memory decay and plasticity stabilization mechanisms, allowing the system to adapt while avoiding catastrophic forgetting.

### 3.4 Cognitive defense loop

**Figure 1:**
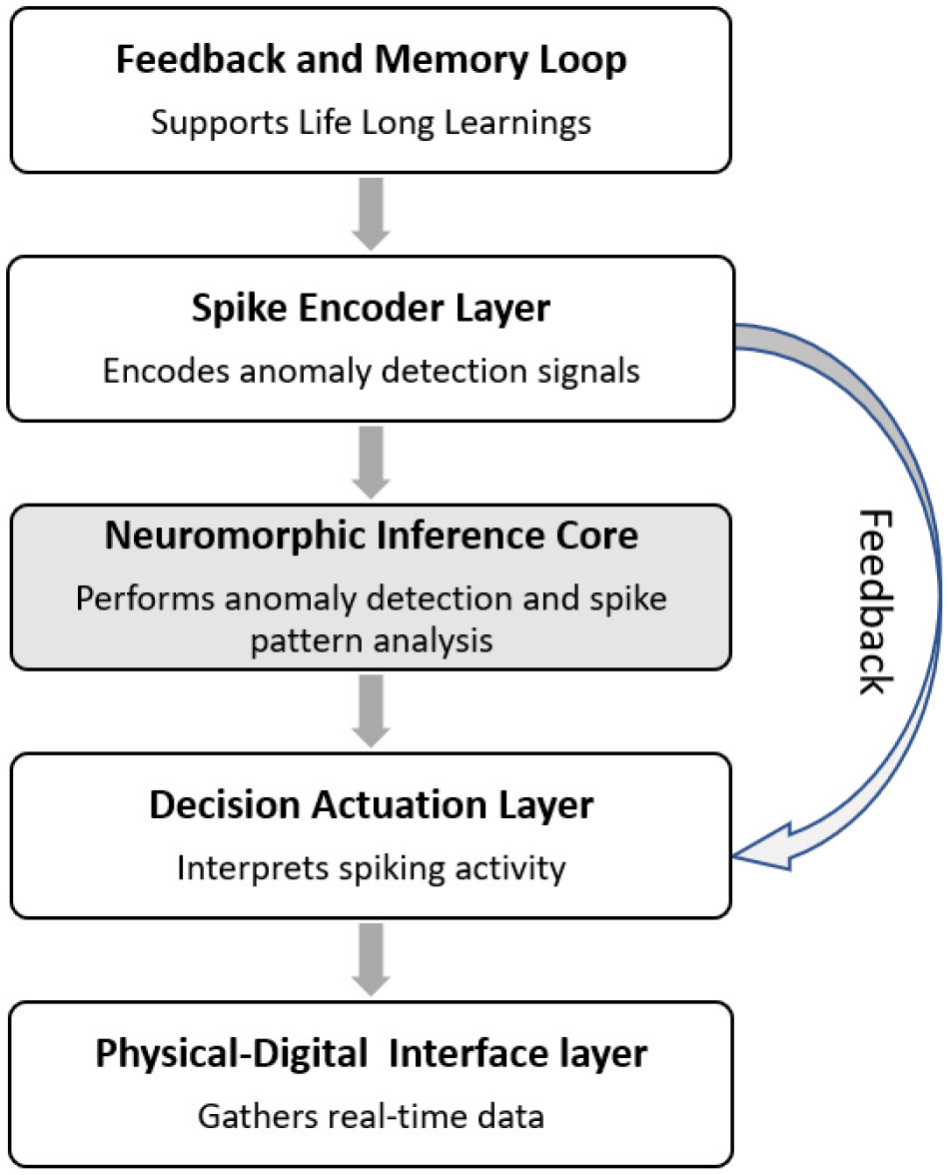
Conceptual framework: the neuromorphic Cyber-Twin. The layered architecture includes a spike encoder, neuromorphic inference core, decision module, physical interface, and feedback loop for adaptive cognitive defense.

Inspired by biological immune and learning systems, the NCT incorporates a cyclical defense loop, as shown in Figure 1. This loop ensures that the NCT is not a static classifier but an evolving system capable of improving its threat recognition over time, especially in adversarial or previously unseen environments.

### 3.5 Comparison with traditional DT security models

Traditional DT security frameworks rely heavily on cloud-based detection, periodic retraining, and often require labeled data. In contrast, the NCT:

- Operates locally with minimal energy overhead,
- Supports unsupervised and continual learning,
- Leverages biologically plausible mechanisms for real-time reasoning.

This architecture positions the NCT as a future-proof framework for intelligent, resilient, and self-defending cyber-physical environments.

## 4 Neuro-cognitive foundations

To justify the biological plausibility and computational design of the NCT, this section outlines the foundational principles of cognitive neuroscience that inspire its core components. We focus particularly on the neural mechanisms that support sparse, event-driven computation and adaptive learning in dynamic environments, drawing parallels to spiking neural networks and plasticity-based memory systems.

### 4.1 Biological inspiration: cognitive security models

**Figure 2:**
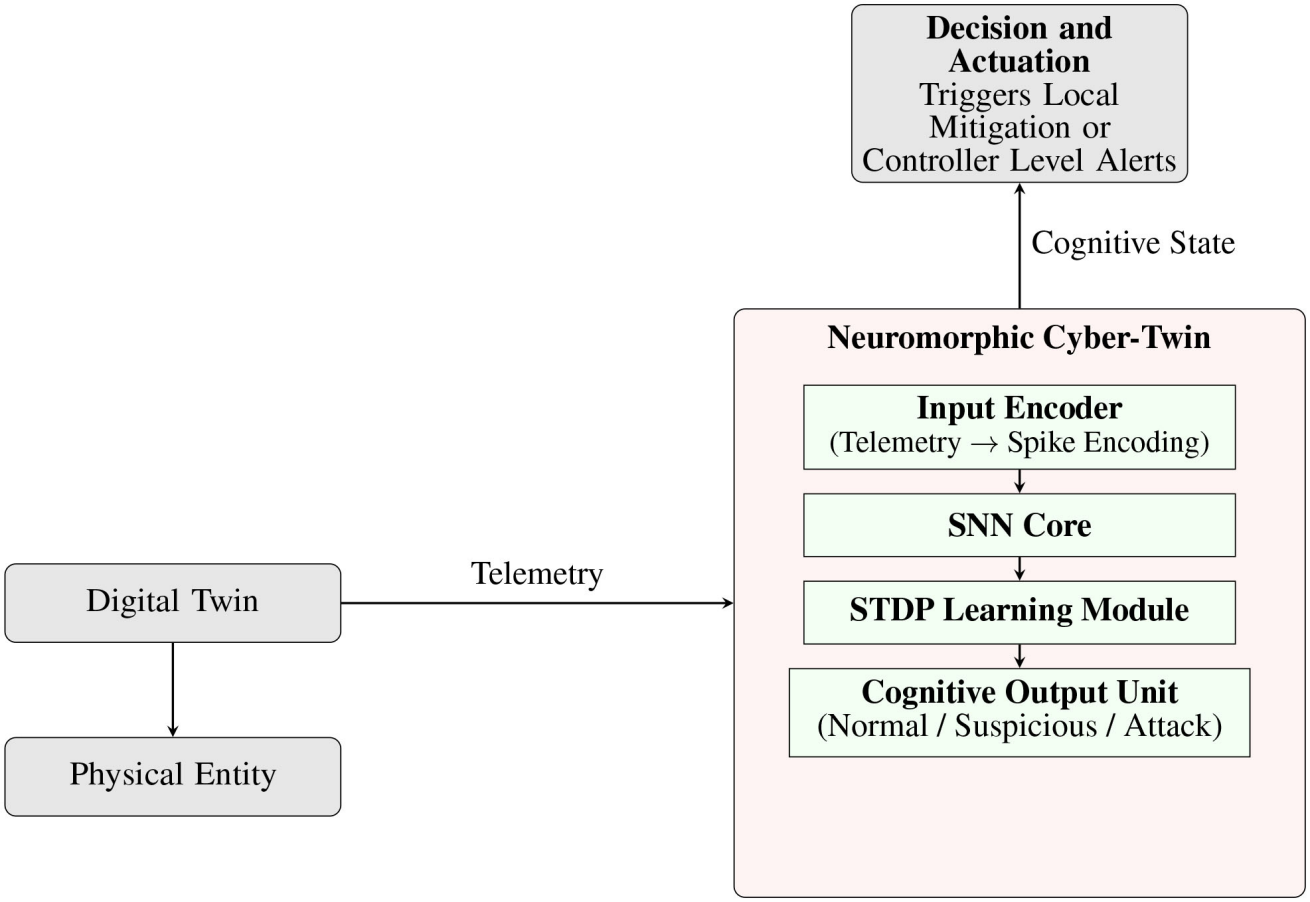
Expanded architecture of the Neuromorphic Cyber-Twin. The telemetry received from the Digital Twin is spike-encoded and processed through an SNN core with embedded STDP learning. The output is a cognitive state used to trigger local or controller-level defense actions.

The NCT is fundamentally inspired by biological neural systems, particularly the brain's ability to process sensory input, adapt to novel threats, and execute defensive actions in real time. The proposed framework mirrors cognitive security behaviors such as attention, recognition, learning, and self-regulation, similar to how the immune and nervous systems co-operate to identify and neutralize external threats. These concepts are translated into computational equivalents via spiking neurons, plastic synapses, and feedback mechanisms, enabling the DT to autonomously detect behavioral anomalies and respond contextually. The layered organization and data-flow of the NCT are illustrated in Figure 2.

### 4.2 Spiking neural networks

SNN represent the third generation of neural models. Unlike conventional artificial neural networks, which rely on continuous values, SNNs operate on discrete spike events across time. Each neuron integrates inputs as membrane potential and fires only when a threshold is reached, emulating real synaptic firing patterns. This mechanism leads to sparse, energy-efficient communication, a critical requirement for embedded or real-time cybersecurity systems.

While the NCT framework conceptually adopts spiking neuron models, the Leaky Integrate-and-Fire (LIF) model is used in preliminary simulations due to its simplicity and suitability for rapid prototyping. However, the architecture remains agnostic to the specific neuron model and can accommodate more complex models (e.g., Izhikevich, Adaptive Exponential) depending on application needs and hardware capabilities. The neuron model in the Leaky LIF model, defined as:


(1) $$\begin{array}{l} \tau_{m}\frac{dV_{m}\left(t\right)}{dt}=-V_{m}\left(t\right)+R_{m}I\left(t\right) \end{array}$$

where *V*_*m*_(*t*) is the membrane potential, *I*(*t*) is the input current, τ_*m*_ is the membrane time constant, and *R*_*m*_ is the membrane resistance. When *V*_*m*_(*t*) exceeds a threshold *V*_*th*_, the neuron emits a spike and resets.

### 4.3 STDP

In the conceptual design of the NCT, learning is envisioned through STDP, a biologically inspired, unsupervised learning rule that enables temporal adaptation and memory formation without the need for labeled data. While STDP aligns well with the NCT's cognitive objectives and supports localized, online learning, the architecture remains agnostic to the specific learning rule. Depending on the target application or neuromorphic hardware, alternative mechanisms such as Reward-Modulated STDP (R-STDP), Hebbian plasticity, or homeostatic adaptation may also be integrated to balance learning stability, context sensitivity, and energy efficiency. For STDP synaptic weights are adjusted based on the temporal correlation between pre-synaptic and post-synaptic spikes:


(2) $$\Delta w=\{\begin{array}{ll} A_{+}e^{-\Delta t/\tau_{+}}, & \text{if }\Delta t>0\\ -A_{-}e^{\Delta t/\tau_{-}}, & \text{if }\Delta t<0 \end{array}$$

where Δ*t* = *t*_*post*_−*t*_*pre*_ is the timing difference between the spikes, and *A*_+_, *A*_−_, τ_+_, τ_−_ are the potentiation and depression constants. This allows the NCT to autonomously adapt to environmental changes and evolving attack patterns without the need for explicit labels or centralized retraining (Izhikevich et al., 2004).

### 4.4 Spike encoding for digital twin telemetry

To enable neuromorphic inference, continuous telemetry streams from the Digital Twin must be transformed into spike trains. This is achieved through biologically inspired encoding strategies. Let *x*(*t*) represent a real-valued feature (e.g., latency, voltage, signal strength) at time *t*, as discussed in Table 1.

**Table 1:** Comparison of spike encoding strategies for digital twin telemetry.

**Encoding strategy**	**Description**	**Advantages / limitations**
Rate coding	Spike frequency is proportional to the input magnitude over a time window.	+ Simple to implement and robust to noise. − Low temporal precision; not ideal for fast-changing or bursty signals.
Latency coding	Input value is encoded as the timing of the first spike, lower inputs spike earlier.	+ High temporal resolution; efficient for rapid event detection. − Susceptible to jitter and time alignment errors.
Rank / threshold coding	Spikes are generated only for top-*k* input values or those exceeding a threshold.	+ Efficient spike usage; highlights salient or dominant features. − May neglect subtle but relevant anomalies.
Burst / binary coding	Spikes encode categorical states using bursts or binary presence.	+ Useful for representing discrete events (e.g., packet type, state transitions). − Less effective for continuous-valued telemetry.

Latency coding has been widely studied for its energy benefits: empirical evaluations show that latency-based encoding can achieve comparable accuracy to rate coding while significantly reducing spike counts and communication overhead, making it highly suitable for resource-constrained neuromorphic deployments (Diehl and Cook, 2015; Mostafa, 2018).

### 4.5 Toward a quantitative evaluation framework

To operationalize the NCT, future work must define a rigorous evaluation methodology. We propose the following roadmap:

- Simulation Platforms:
  - *Brian2* – for spiking simulation and rapid prototyping of STDP-based SNNs.
  - *Nengo* – for higher-level cognitive models with visualization and encoding utilities.
  - *Loihi SDK / Lava* – for benchmarking event-driven neuromorphic inference on Intel's hardware.
- Suggested Evaluation Metrics:
  - *Spike Count per Class:* Sparsity and discriminability of spike patterns across nominal vs. anomalous states.
  - *False Positive/Negative Rate:* Accuracy of anomaly detection under temporal and contextual drift.
  - *Plasticity Saturation:* Monitoring weight convergence and forgetting during continuous learning.
  - *Latency to React:* Time from anomaly onset to defense policy activation.
  - *Energy per Inference:* Estimated using SNN hardware counters or runtime profiling.

In future benchmarks, these metrics can be tracked under varying attack types (e.g., spoofing, delay injection) and environments (smart grid, V2X, medical twins). Synthetic DT datasets or real telemetry logs can serve as inputs for performance profiling.

A range of spike encoding methods, including rate coding, latency coding, and burst coding, were considered in this study. Rate coding offers implementation simplicity but suffers from higher energy costs due to dense spiking activity, whereas latency coding provides sparse, energy-efficient representations at the expense of timing precision. Burst coding enhances robustness in noisy environments but increases decoding complexity. Comparative evaluations in prior works, such as Roy et al. (2019); Schuman et al. (2022) demonstrate that hybrid approaches can balance accuracy and power consumption, informing our choice of latency coding for anomaly detection tasks where energy efficiency and responsiveness are critical. The proposed evaluation metrics, including detection latency, spike sparsity, energy per inference, and throughput, are intended as design guidelines for future benchmarking rather than results reported here. These metrics emphasize neuromorphic advantages such as low-latency inference, sparse computation, and power efficiency, serving as a consistent baseline for subsequent large-scale validation across hardware and simulator platforms.

For transparency, we provide the implementation and demonstration results via GitHub. On the NSL-KDD dataset, our prototype SNN achieved lower accuracy and F1-scores than conventional ML baselines. This is expected in a conceptual study, where the emphasis lies on demonstrating the feasibility of scaling, real-time Digital Twin integration, and neuromorphic efficiency (latency, sparsity, energy). These results are therefore presented as illustrative evidence rather than optimized benchmarks, pointing toward directions for future refinement.

## 5 Use cases and operational scenarios

The NCT is designed for intelligent, autonomous defense across a variety of cyber-physical systems where traditional security models are insufficient. This section outlines several realistic domains where the NCT framework can be deployed to enhance resilience and cognitive responsiveness.

### 5.1 Smart grids and industrial automation

Digital Twins in smart grid environments model physical infrastructure such as substations, transformers, and load balancing systems. These assets are frequent targets for coordinated cyberattacks, including load-altering or false data injection. The NCT can monitor voltage fluctuations, response latencies, and operational commands in spike-encoded format. SNNs can detect spatiotemporal anomalies and initiate corrective actions, such as isolating a subnetwork or reversing a control instruction, before failures spread (Lu et al., 2020).

### 5.2 Autonomous vehicles and V2X communication

Vehicular Digital Twins replicate the dynamic states of connected vehicles, enabling predictive control and fleet-wide optimization. However, V2X systems are vulnerable to spoofing, jamming, or man-in-the-middle attacks. By embedding neuromorphic modules into vehicle DTs, the NCT can monitor real-time telemetry (e.g., speed, heading, latency) and recognize temporal inconsistencies that deviate from learned traffic behavior. Upon detection, the NCT may trigger emergency stop routines or reroute communications to verified peers (Xu et al., 2024).

### 5.3 Healthcare and medical device security

Medical Digital Twins represent patient vitals, infusion pumps, and implant telemetry. Given their safety-critical nature, these systems require zero-latency and zero-failure cybersecurity. A neuromorphic security layer can monitor patterns in heart rate, signal delay, and dosage cycles. Temporal learning via STDP allows the NCT to adapt to personalized baselines, identifying anomalies in device activity, network integrity, or patient response, even with limited data Al-Dalati (2023).

### 5.4 Federated smart city infrastructure

In smart cities, federated DTs govern public assets, such as traffic systems, surveillance cameras, or utility grids, in multiple jurisdictions. These systems must function under noisy, distributed, and adversarial conditions. The NCT framework enables localized threat detection and response at the DT node level without depending on centralized data fusion. As a result, security decisions become context-sensitive and computationally autonomous, ensuring system-wide scalability and robustness (Kreuzer et al., 2024).

### 5.5 Comparison with classical detection systems

As shown in Table 2, the proposed NCT offers significant advantages over classical detection systems, particularly in environments that demand ultra-low latency, high adaptivity, and energy-efficient cyber defense. By leveraging event-driven spiking neural networks and unsupervised plasticity mechanisms, the NCT can operate at the edge with minimal computational overhead while continuously adapting to emerging threats. This makes it particularly suited for dynamic and resource-constrained cyber-physical systems such as autonomous vehicles, industrial IoT, and smart healthcare infrastructures.

**Table 2:** Comparison of classical and neuromorphic DT security.

**Feature**	**Classical AI/ML**	**NCT (SNN-based)**	**References**
Learning type	Supervised / offline	Unsupervised / online	Nazari and Amiri, 2025; Daddinounou et al., 2025
Latency sensitivity	Medium to high	Ultra low	Chen et al., 2025; Qu et al., 2025; Sun et al., 2025
Energy efficiency	Moderate to high	High (event-driven)	Liu et al., 2024; Sun et al., 2025
Adaptivity to drift	Weak (requires retraining)	Strong (via STDP)	Chakraborty and Mukhopadhyay, 2021; Demin et al., 2021
Deployment layer	Cloud / edge	Edge / On-device	Cañete et al., 2022; Davies et al., 2021
Explainability	Moderate	Low to moderate	Iaboni and Abichandani, 2024; Stadtmann et al., 2023

## 6 Challenges and research directions

Despite its promising architecture, the implementation of the NCT presents several technical and interdisciplinary challenges. Addressing these issues is critical for advancing from conceptual design to deployable systems. Summary of key challenges is tabulated in Table 3.

**Table 3:** Summary of key technical and research challenges.

**Challenge**	**Proposed direction**
Spike encoding from DT telemetry	Design of adaptive hybrid spike encoders (rate-based + latency-based) for noisy and dynamic telemetry signals.
Real-time SNN integration	Development of lightweight middleware and on-device simulators for seamless integration with DT platforms.
Evaluation frameworks	Creation of DT-specific benchmarks combining anomaly detection metrics with cognitive adaptability scores.
Hardware deployment	Porting to neuromorphic chips (e.g., Intel Loihi, BrainChip Akida) and edge co-processor integration.
Interdisciplinary gaps	Toolkits and design workflows to support co-evolution of DT models and SNN architectures across domains.

### 6.1 Neuromorphic-DT integration

Integrating SNNs with Digital Twin platforms is non-trivial. Most DT frameworks are developed in high-level programming environments (e.g., MATLAB, Unity, or Python-based simulators), while neuromorphic systems typically run on specialized hardware or simulators (e.g., Brian2, Nengo, Intel Loihi). Bridging these domains requires middleware capable of real-time, spike-based communication, along with standardized APIs and data encoders (Muir and Sheik, 2025).

### 6.2 Data encoding and representational fidelity

Encoding continuous telemetry streams from the DT into spike trains is a major bottleneck. Rate coding is simple but may lose critical temporal detail; latency coding is sensitive but difficult to calibrate. Choosing the appropriate encoding method remains an open problem and likely requires dynamic switching strategies based on context, feature dimensionality, and real-time constraints (Auge et al., 2021).

### 6.3 Evaluation metrics for cognitive security

Unlike traditional security systems that evaluate performance using accuracy or precision-recall, cognitive systems like the NCT need novel metrics. These include:

- *Cognitive latency:* Time to recognize and react to a novel threat.
- *Plasticity-efficiency balance:* Ability to learn continuously without catastrophic forgetting.
- *Adaptability index:* Performance under concept drift or adversarial perturbation.

Developing benchmark datasets and simulators that reflect DT-specific anomalies is equally essential.

### 6.4 Hardware constraints and scalability

While neuromorphic processors (e.g., Loihi, Akida, BrainScaleS) offer impressive energy efficiency, they are still in early adoption phases. Their availability, documentation, and development toolchains remain limited. Furthermore, scaling NCTs across thousands of DTs (e.g., in a smart city) requires distributed learning protocols, federated update mechanisms, and secure SNN sharing frameworks (Daddinounou et al., 2025).

### 6.5 Interdisciplinary knowledge gaps

The realization of NCTs demands expertise across domains: neuromorphic computing, DT simulation, cybersecurity, and embedded systems. However, current development workflows are siloed. Collaborative research platforms, cross-domain training, and integrated toolkits will be vital for success (Kreuzer et al., 2024).

### 6.6 Challenges and strategies in federated STDP aggregation

Federated Spiking Neural Networks (SNNs) with STDP learning face unique challenges due to their local, time-dependent updates and asynchronous behavior. To maintain global learning consistency while preserving node-specific adaptability, we propose the following strategies:

1) Avoiding Catastrophic Forgetting: Instead of directly overwriting local synapses, the aggregator performs *weighted aggregation of synaptic deltas*:

(3) $$\begin{array}{l} w_{i}^{\left(t+1\right)}=w_{i}^{\left(t\right)}+\frac{1}{N}\overset{N}{\underset{k=1}{\sum }}\Delta w_{i}^{\left(k\right)} \end{array}$$

Here, *w*_*i*_ is the synapse of interest and $\Delta w_{i}^{\left(k\right)}$ is the local STDP-induced change at device *k*.
2) Temporal Alignment of Spike Events: To resolve drift or desynchronization across devices, local spike updates are:
  - *Timestamped* using quantized intervals (e.g., 1 ms slots),
  - *Aligned* using a shared logical clock or synchronization pulses via DT middleware.
3) Privacy-Preserving Learning: Before transmitting synaptic deltas to the aggregator, each NCT applies:
  - *Gradient clipping*: Δ*w*←min(Δ*w*, τ)
  - *Differential privacy noise*: $\Delta w\leftarrow \Delta w+N\left(0,\sigma^{2}\right)$

This ensures that no individual input pattern can be inferred from shared weight updates.

Federated learning in neuromorphic systems introduces unique challenges compared to conventional federated averaging, primarily due to the temporal and non-IID nature of spiking activity across distributed nodes. In the proposed federated STDP framework, synaptic weight updates are computed locally via STDP and periodically aggregated using spike-derived delta weights, as suggested in Algorithm 1. Literature on distributed neuromorphic learning (Wang et al., 2023) highlights trade-offs between communication frequency, convergence stability, and hardware efficiency, motivating an adaptive aggregation schedule driven by spike activity drift rather than fixed update intervals. Summary of key research challenges have been discussed in Table 3.

**Algorithm 1:** Federated STDP Aggregation with Privacy and Temporal Alignment.

**1**. For each round *t* = 1, 2, …
**2**. For each NCT device *k*∈{1, …, *N*} (in parallel): • Collect telemetry and encode into spike trains. • Run local STDP updates on weights *W*_*k*_. • Compute $\Delta W_{k}=W_{k}^{\left(t\right)}-W_{k}^{\left(t-1\right)}$. • Align STDP traces via time bins. • Apply clipping: Δ*W*_*k*_←min(Δ*W*_*k*_, τ). • Add Gaussian noise: $\Delta W_{k}\leftarrow \Delta W_{k}+N\left(0,\sigma^{2}\right)$. • Transmit Δ*W*_*k*_ to aggregator.
**3**. Aggregator computes global model: $W^{\left(t+1\right)}\leftarrow W^{\left(t\right)}+\frac{1}{N}\underset{k}{\sum }\Delta W_{k}$
**4**. Broadcast updated global weights *W*^(*t*+1)^ to all NCT nodes.

Parameter tuning of STDP plays a critical role in achieving stable learning. Excessively large potentiation or depression rates lead to weight saturation, while conservative values slow convergence. We followed stability ranges reported in (Davies et al., 2021; Yan et al., 2019), setting the learning rates in a range that preserves spike-timing sensitivity while avoiding synaptic saturation. These considerations ensure that the model remains energy-efficient and robust in dynamic Digital Twin environments.

Adjustable Parameters In addition to the strategies above, two further considerations improve robustness in practice. First, aggregation frequency can be made adaptive, triggered by spike distribution drift thresholds rather than fixed intervals, thereby reducing communication overhead. Second, asynchronous update handling mechanisms, as explored in distributed FL systems, can be integrated to tolerate stragglers and heterogeneous DT environments.

## 7 Benefits and limitations

**Figure 3:**
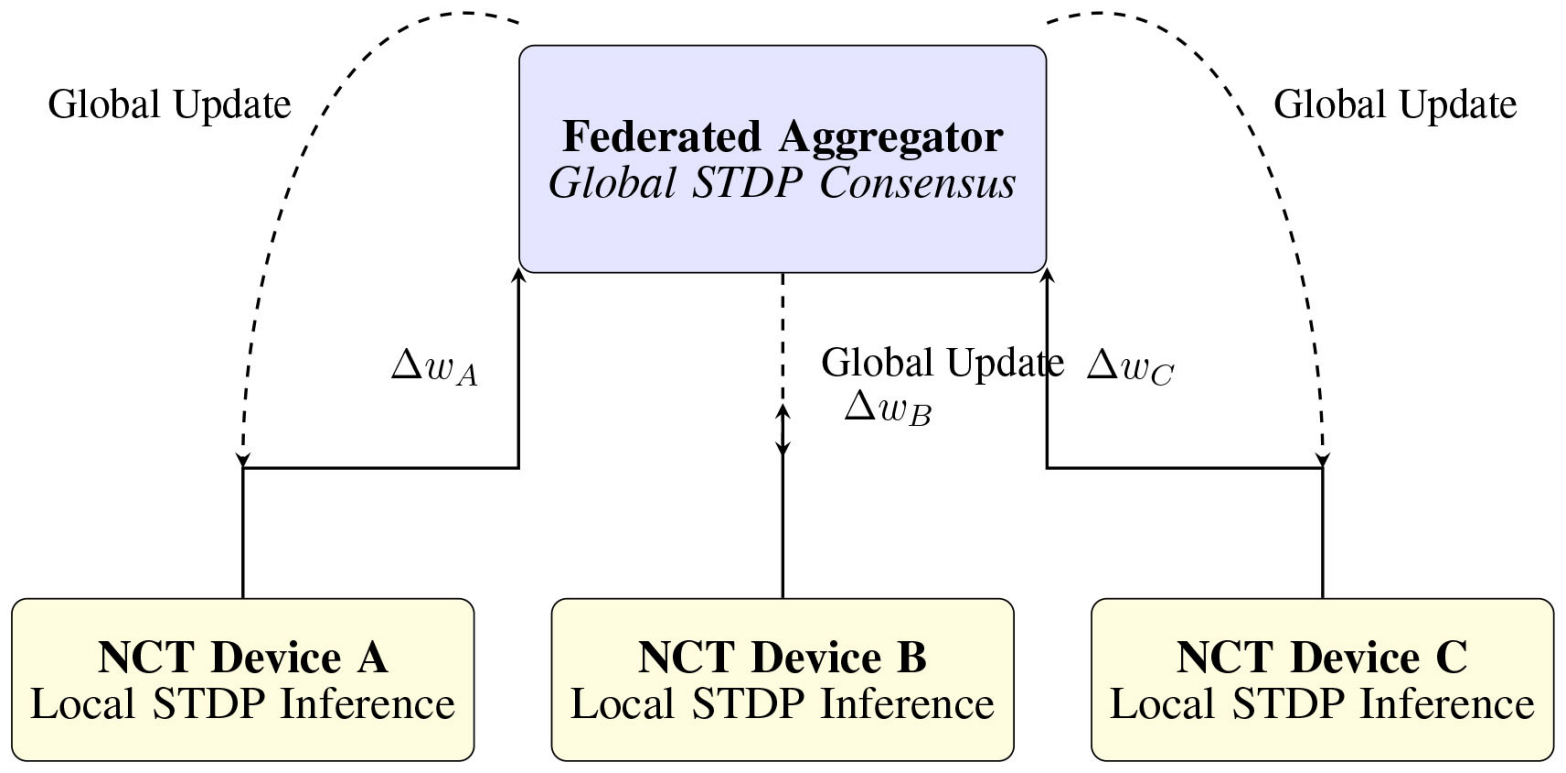
Federated learning flow for neuromorphic Cyber-Twins. Local STDP-based inference produces synaptic deltas (Δ*w*), which are aggregated to form a consensus model, redistributed to edge NCTs.

While the NCT presents a promising approach to embedding cognitive adaptability within Digital Twin security architectures, it is essential to assess both its strengths and inherent trade-offs. In this section, we outline the key advantages that differentiate the NCT from traditional systems, while also acknowledging current limitations related to scalability, interpretability, and hardware readiness. The overall federated STDP aggregation workflow with temporal alignment is shown in Figure 3.

### 7.1 Key advantages

The NCT presents several compelling advantages over traditional DT security architectures:

- Low-latency inference: The use of event-driven SNN allows the NCT to respond to threats with minimal processing delay, which is critical for time-sensitive cyber-physical environments (Datta et al., 2025).
- Online adaptivity: The STDP-based learning mechanism enables continuous, unsupervised adaptation to novel or evolving threats without requiring retraining or human intervention.
- Energy efficiency and scalability: SNNs inherently offer low-power computation due to sparse, asynchronous activation, making the NCT suitable for deployment on edge and embedded neuromorphic hardware platforms.
- Independence from cloud infrastructure: By processing locally on the DT or associated edge node, the NCT reduces dependence on centralized cloud analytics, thereby improving latency, resilience, and data privacy.

### 7.2 Current limitations

Despite its theoretical promise, the realization of the NCT model presents several limitations and open challenges:

- Lack of domain-specific datasets: There is a notable scarcity of publicly available Digital Twin telemetry datasets with annotated cyber anomalies, hindering standardized training and evaluation.
- Interpretability of spiking decisions: While biologically plausible, the internal decision-making process of SNNs, especially in STDP-based architectures, remains difficult to explain or audit, limiting user trust in critical infrastructure settings.
- Hardware support and deployment readiness: Neuromorphic processors (e.g., Intel Loihi, BrainChip Akida) are still under development, and integration with mainstream Digital Twin platforms (e.g., Unity, Siemens NX, or ANSYS Twin Builder) remains limited.

Addressing these limitations will require advances in neuromorphic interpretability, standardized telemetry encoding interfaces, and co-development of datasets and toolchains for hybrid DT–SNN platforms.

### 7.3 Federated cognitive twins: toward distributed neuromorphic defense

As Digital Twin ecosystems scale across smart cities, industrial zones, and vehicular networks, the centralized processing of cyber-defense data becomes infeasible due to latency, privacy, and scalability constraints. To address this, we envision a future class of *Federated Cognitive Twins (FCTs)*, a distributed, privacy-preserving neuromorphic framework where each Digital Twin hosts a localized instance of the NCT, collaboratively learning through federated mechanisms.

Each FCT unit is equipped with its own spike-based learning module, processing local telemetry in real time using SNN-based inference. Periodically, learned synaptic updates or anomaly signatures are aggregated across a network of twins via secure, federated communication protocols. Unlike conventional federated learning, which transmits large ANN weights or gradients, FCTs can exchange lightweight synaptic delta patterns or compressed spiking templates, significantly reducing bandwidth and preserving spatio-temporal data locality.

- Decentralized Learning: Cognitive twins update their own SNNs using STDP or other local rules and contribute only sparse summaries to a global model.
- Privacy-Aware Security Intelligence: Raw telemetry or sensitive behavioral data never leaves the local DT node, addressing regulatory and ethical concerns.
- Federated Anomaly Consensus: Anomalies detected by one twin can inform the response policies of others, enabling swarm-like situational awareness across a distributed CPS.
- Resilience to Partitioning: Even in cases of network segmentation or link failure, FCTs maintain their cognitive defense loop independently, ensuring continued operation and gradual convergence when reconnected.

This approach is especially suited to scenarios such as:

- Smart grids with regional substations and DTs per transformer,
- Vehicular fleets in V2X environments with onboard neuromorphic cores,
- Hospital networks where each medical device or patient DT acts as an autonomous anomaly detector.

The development of FCTs will require innovations in spike-based federated learning protocols, distributed SNN coordination, and neuromorphic model compression. Future work may explore the design of lightweight consensus algorithms that operate over temporal spiking patterns and enable secure, explainable collaboration among autonomous Digital Twins.

### 7.4 Synthetic telemetry for neuromorphic evaluation

Given the lack of publicly available telemetry datasets with labeled anomalies in Digital Twin systems, we propose a synthetic data generation strategy to evaluate the Neuromorphic Cyber-Twin (NCT) pipeline under controlled yet realistic conditions. Table 4 summarizes representative anomalies, suitable encoders, and aligned NCT use cases.

1) Domain-Specific Simulators: We suggest leveraging domain-specific simulators to generate deterministic, controllable system behaviors:
  - GridLAB-D: For generating voltage, frequency, and load profiles in smart grids.
  - CARLA: For simulating vehicular dynamics and inter-vehicle coordination in autonomous driving scenarios.
  - NS-3: For network traffic flow, jitter, and congestion telemetry.
2) Anomaly Injection Framework: Time-series outputs from these simulators can be perturbed using known anomaly types such as:
  - *Gaussian Noise*: For simulating sensor degradation.
  - *Step Discontinuities*: For power/load switching anomalies.
  - *Temporal Drift*: For clock desynchronization or latency buildup.
3) Spike Encoding and STDP Evaluation: These time-series can then be encoded using rate or latency coding schemes and fed into SNN simulators such as *Brian2* or *Nengo*. STDP-based learning dynamics can be assessed for anomaly recognition accuracy, convergence speed, and spike sparsity.

**Table 4:** Synthetic Anomalies and Corresponding Spike Encoding Strategies.

**Anomaly type**	**Encoding strategy**	**Target use case**
Gaussian noise	Rate coding	Sensor degradation in smart metering systems
Step jump (Abrupt load change)	Time-to-first spike	Grid overload detection or sudden cyber-physical attacks
Latency drift	Latency coding with thresholding	Clock desynchronization in V2X communications
Packet dropout	Binary spike encoding	Communication failure in vehicular coordination protocols
Periodic burst	Multi-spike burst coding	Detection of Denial-of-Service (DoS) or spoofing attempts

### 7.5 Prototype simulation: STDP-based cognitive response with synthetic telemetry

To assess the feasibility of the proposed NCT framework, we developed a proof-of-concept prototype using the Brian2 spiking neural network simulator. The prototype models a network of approximately 100 excitatory and 25 inhibitory neurons configured with biologically inspired synaptic dynamics and spike-timing dependent plasticity. Synthetic telemetry signals are generated to emulate normal and anomalous system behaviors, which are then encoded into spike trains using a Poisson-based event-driven strategy, as suggested in Algorithm 2.

**Algorithm 2:** STDP Simulation Pseudocode for NCT

**1**. Initialize Brian2 simulation environment.
**2**. Generate synthetic telemetry and encode into spike trains (rate/latency coding).
**3. For each NCT node:**
a. Define LIF neuron population (e.g., ~100 excitatory + 25 inhibitory).
b. Connect input layer to neurons via STDP synapses.
c. Apply Hebbian STDP rule: $\begin{array}{l} \Delta w=A_{+}e^{-\Delta t/\tau_{+}}\;\text{(potentiation)}\\ \Delta w=-A_{-}e^{\Delta t/\tau_{-}}\;\text{(depression)} \end{array}$
d. Run simulation for duration *T*.
e. **If** anomaly is injected → observe potentiation in specific pathways.
f. **Else** → weights stabilize with sparse updates.
**4**. Record simulation outputs: Spike raster plot, Membrane voltage trace, Synaptic weight evolution, Inference latency, spike sparsity, runtime statistics, Memory usage, and STDP iteration count.

This setup allows us to observe how spiking neurons adaptively separate benign from anomalous activity streams. Key performance indicators proposed earlier in Section IV-E, such as inference latency, spike sparsity, and runtime efficiency, were measured. Initial trials showed inference latency on the order of ~0.5 ms per input sample, spike sparsity around 15%, and an average runtime of ~4 seconds for processing 50 samples on a standard workstation. While intentionally limited in scale, these results demonstrate that the NCT architecture can operationalize the proposed evaluation metrics even in a lightweight prototype. The core simulation workflow is summarized in the following pseudocode, which outlines the main steps of the implementation pipeline. The full prototype, including simulation scripts, synthetic telemetry generators, and visualization routines, is available as a Jupyter notebook in the open repository referenced in the Code and Data Availability section. This ensures that the prototype results reported here can be reproduced, while larger-scale benchmarking with real-world cybersecurity datasets (e.g., CICIDS2017, UNSW-NB15, IoT-23) and comparisons with conventional IDS/ML models remain key directions for future work.

## 8 Discussion and research outlook

Although this work presents a novel conceptual architecture, several extensions are proposed to enhance its validation and alignment with emerging trends in computational intelligence. This work contributes to the broader advancement of neuromorphic intelligence for secure, low-power, and adaptive computing in distributed environments. The proposed Neuromorphic Cyber-Twin architecture builds upon current trends that emphasize energy-efficient, cognitive processing using spiking neural networks (SNNs). Recent studies have proposed federated STDP-based frameworks that enable collective adaptation across edge devices by sharing synaptic deltas, demonstrating scalable neuromorphic learning across networked systems (Wang et al., 2023). In parallel, hierarchical spiking architectures have been applied to IoT domains for detecting spatiotemporal anomalies using biologically inspired mechanisms (Rathi et al., 2023). This work extends these developments by embedding neuromorphic inference directly into the Digital Twin ecosystem, enabling context-aware threat perception and adaptive response in virtualized cyber-physical environments.

### 8.1 Prototype simulation and neuromorphic toolchains

Future iterations of the NCT will benefit from implementation in open-source neuromorphic simulation platforms. Among the most promising are:

- Brian2: A Python-based simulator ideal for modeling spiking neuron dynamics, STDP learning, and multiscale experimentation. It allows high configurability for testing spike encoding, adaptation, and cognitive inference under DT-like telemetry sequences.
- Intel Loihi (via Lava SDK): A hardware-aligned platform supporting real-time SNN inference and energy monitoring. Mapping the NCT's modular blocks (input encoder, SNN core, STDP learning) onto Loihi cores will validate latency and power assumptions critical for edge deployments.
- Nengo: Offers abstraction layers for integrating neural models with sensor data, including preliminary Digital Twin integration for robotics and CPS simulations.

**Figure 4:**
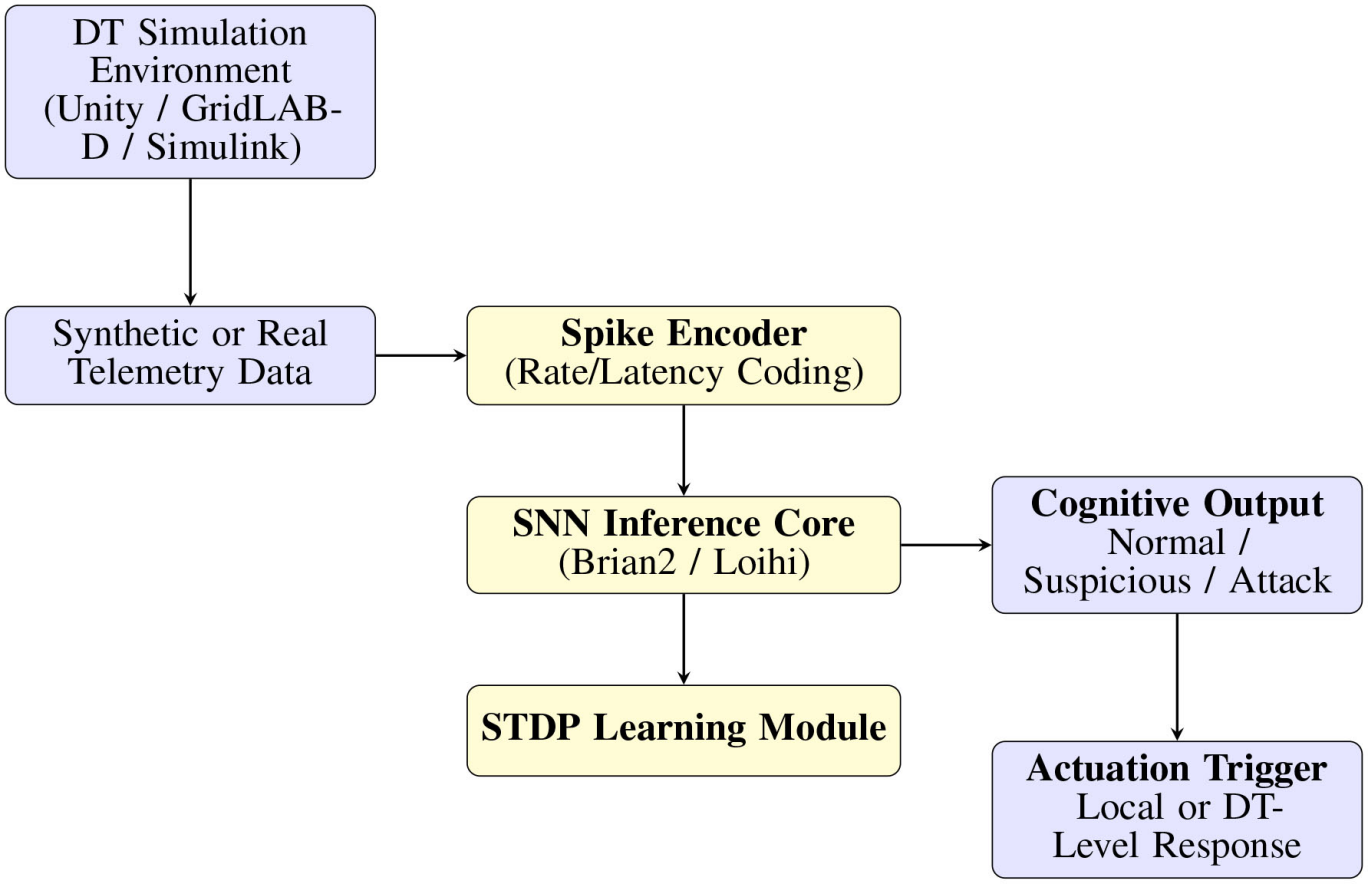
Simulation flow integrating Digital Twin telemetry with neuromorphic inference using spike encoders, SNN cores (Brian2/Loihi), and STDP-based learning.

A conceptual implementation roadmap is illustrated in Figure 4, outlining the flow from telemetry data collection (via DT simulation tools like Unity or GridLAB-D) to neuromorphic inference via Brian2/Loihi.

### 8.2 Platform selection guidance for NCT prototyping and deployment

Selecting the appropriate neuromorphic platform is critical to the implementation of the Neuromorphic Cyber-Twin (NCT), depending on the development stage, performance priorities, and available resources. Below, we summarize when each platform is best suited for research, prototyping, and deployment:

- Brian2 is most suitable for early-stage prototyping, where fine-grained control over spike encoding, plasticity behavior, and event timing is necessary. It allows detailed experimentation with STDP, LTP, and latency-based encoding without hardware constraints.
- Loihi (v2) is ideal for real-time, energy-aware inference and deployment on edge neuromorphic hardware. Once the NCT model is validated in simulation, it can be compiled and tested on Loihi for timing, power, and adaptive responsiveness.
- Nengo provides flexibility across the stackfrom algorithm design to embedded deployment. It supports high-level architecture definition with optional backends (e.g., Loihi or FPGA), making it suitable for hybrid workflows.
- SpiNNaker is recommended for large-scale cognitive twin networks where distributed inference is required across multiple NCT instances (e.g., federated edge-twin clusters in smart grids or autonomous fleets).
- Lava is a developing but promising framework that unifies neuromorphic software development for Intel's Loihi platform. It is suited for researchers aiming for full-stack alignment with future neuromorphic ecosystems.
- BindsNET is appropriate for machine learning researchers wishing to integrate SNN-like behavior into deep learning pipelines. It offers STDP modeling with PyTorch compatibility and is useful for comparing ANN vs. SNN in anomaly detection or behavior prediction tasks.

The choice of platform may evolve across the lifecycle of the NCT, from simulation (Brian2) to deployment (Loihi/Lava) and scaling (SpiNNaker). This modularity in toolchain enables iterative development with minimal architecture rework. While neuromorphic platforms such as Intel's Loihi Hala Point and BrainChip Akida demonstrate significant improvements in scalability and energy efficiency, they primarily target edge inference or general-purpose spiking workloads rather than a tightly integrated cybersecurity–Digital Twin (DT) pipeline. The proposed Neuromorphic Cyber-Twin (NCT) distinguishes itself by unifying telemetry-driven encoding, cognitive-layer adaptation, and federated STDP updates within a single conceptual framework. Whereas Hala Point emphasizes massive scalability (over 1.15B neurons) and Akida prioritizes ultra-low-power edge anomaly detection, NCT focuses on a hierarchical, DT-aware control loop, enabling distributed cyber-physical threat detection and on-device learning. This integration of SNN-based adaptation with DT orchestration and federated weight aggregation fills a gap not explicitly addressed by existing neuromorphic hardware or edge-AI deployments.

### 8.3 Practical implementation trade-offs

While the Neuromorphic Cyber-Twin (NCT) framework presents a conceptually robust and biologically inspired architecture for secure, adaptive DT environments, its real-world implementation introduces several practical challenges. These must be carefully considered to transition from simulation-based validation to deployment in operational settings. Comparison of neuromorphic platforms and tools for NCT development are tabulated in Table 5.

**Table 5:** Comparison of neuromorphic platforms and tools for NCT development.

**Platform**	**Type**	**Real-time execution**	**Hardware acceleration**	**Plasticity support (e.g., STDP)**	**Learning rule support**	**IoT/Edge suitability**
Loihi (v2)	Neuromorphic chip	✓	✓	✓ (STDP, R-STDP)	On-chip adaptive learning	✓
SpiNNaker	Hardware system	✓	✓ (ARM cores)	✓ (event-driven)	Local STDP	× (lab scale)
Brian2	Software simulator	×	×	✓ (custom Python)	Custom STDP models	×
Nengo	Framework + frontend	✓ (via hardware)	✓ (Loihi/FPGA)	✓ (customizable)	Rule-agnostic backend	✓
Lava	Software framework	✓ (with Loihi)	✓	✓ (STDP modules)	Modular APIs	✓
BindsNET	PyTorch library	×	× (CPU/GPU)	✓ (via PyTorch)	Backprop + STDP	×
Neurogrid	Mixed-analog system	✓	✓ (analog accel.)	✓ (Hebbian)	Hebbian + Inhibition	✓
TrueNorth	Digital ASIC	✓	✓	× (no on-chip learning)	Pre-trained only	✓
NSoC (Tianjic)	Hybrid SoC	✓	✓	✓ (mixed-mode STDP)	Hybrid ANN+SNN control	✓
Dynap-SE	Mixed-signal SNN Chip	✓	✓ (event-based)	✓ (AER, Hebbian)	On-chip STDP	✓
BrainScaleS-2	Analog neuromorphic system	✓	✓ (sub-ms analog)	✓ (bio-realistic)	Hebbian, LTP/LTD	× (lab setup)
GeNN	GPU-accelerated SNN framework	✓ (CUDA)	✓ (NVIDIA GPUs)	✓ (custom)	STDP, Izhikevich	× (GPU required)
CARLsim	Software SNN simulator	×	×	✓ (scalable)	STDP, homeostatic	×
NeuroSim	Modeling framework	×	✓ (memristor sim)	✓ (device-level)	STDP, RRAM models	✓ (design-phase)

#### 8.3.1 Hardware deployment readiness

The availability and maturity of neuromorphic hardware remain significant bottlenecks. Although platforms such as *Intel Loihi, BrainChip Akida*, and *SpiNNaker* have demonstrated promising results in event-driven computation, they are still in the early stages of mainstream adoption. Current limitations include:

- Restricted access and documentation: Loihi 2 is not yet widely available for commercial prototyping, and development still requires specialized knowledge and controlled environments (e.g., Lava SDK).
- Toolchain compatibility: Simulators like *Brian2* and *Nengo* offer robust modeling but lack streamlined pipelines for neuromorphic hardware integration.
- Limited edge-ready deployment: Although Akida and Loihi support energy-efficient inference, packaging and interfacing with embedded DT systems remains non-trivial.

A hybrid strategy using general-purpose CPUs to simulate SNN dynamics with progressive hardware migration may support early-stage deployment.

#### 8.3.2 Middleware and API challenges

A critical barrier to practical integration lies in the absence of standardized middleware for spike-based communication between DT platforms and neuromorphic engines. Common DT environments, such as *Unity, MATLAB/Simulink*, and *ANSYS Twin Builder*, are typically time-stepped or event-based simulators, not natively compatible with SNN dynamics.

Key integration challenges include:

- Real-time encoding overhead: Translating multivariate telemetry (e.g., voltage, latency, throughput) into biologically inspired spike trains in real time requires efficient encoders.
- Clock synchronization issues: SNNs depend on temporally precise spike timing, which can be desynchronized in high-level DT environments.
- Interfacing complexity: Bridging event-driven neuromorphic logic with traditional communication interfaces (e.g., REST APIs, MQTT) demands middleware capable of real-time translation and adaptation.

Emerging tools such as *Lava* and *ROS-integrated Brian2 interfaces* may support this gap in the near future, but current solutions are largely bespoke.

#### 8.3.3 Real-world telemetry datasets

An ongoing limitation for validation and benchmarking is the scarcity of publicly available Digital Twin telemetry datasets with labeled cyber anomalies. Compared to classical cybersecurity datasets (e.g., CICIDS, UNSW-NB15), DT telemetry is often:

- Domain-specific: Tailored to verticals such as smart grids, autonomous vehicles, or healthcare.
- Unlabeled or proprietary: Due to privacy, regulation, or intellectual property concerns.
- Non-standardized: Lacking common formats and annotation schemes across industries.

We propose two complementary strategies:

1. Synthetic data generation using simulators like *GridLAB-D* (energy), *CARLA* (automotive), and *NS-3* (networking) with anomaly injection.
2. Collaborative curation of telemetry datasets with industry and regulatory sandboxes, with privacy-preserving techniques for anonymization and sharing.

In the long term, efforts toward open, anonymized telemetry repositories, analogous to *MIMIC-III* in healthcare, would significantly benefit neuromorphic cybersecurity research.

While this work focuses on demonstrating adaptive anomaly detection and federated neuromorphic learning, adversarial robustness remains an important direction for future research. Neuromorphic systems, like traditional deep learning models, can be vulnerable to adversarial perturbations or carefully crafted telemetry signals. Emerging countermeasures such as noise injection, spike jitter regularization, and synaptic weight smoothing (Schuman et al., 2022) will be incorporated into the NCT framework to harden it against adversarial evasion attempts, particularly in safety-critical cyber-physical deployments.

### 8.4 Future benchmarking roadmap

To transition the Neuromorphic Cyber-Twin (NCT) framework from concept to deployable technology, we propose a systematic benchmarking plan:

- Simulation Environments: Utilize CARLA for autonomous driving scenarios, GridLAB-D for power grid Digital Twins, and NS-3 for communication network emulation to create realistic telemetry streams.
- Cybersecurity Datasets: Integrate standard datasets such as CICIDS2017, UNSW-NB15, and IoT-23 to train and evaluate intrusion detection and anomaly classification accuracy.
- Hardware Benchmarking: Deploy the NCT architecture on neuromorphic hardware platforms (Intel Loihi 2, BrainChip Akida, SpiNNaker) to measure power efficiency, spike sparsity, and inference latency at scale.
- Federated and Distributed Validation: Test the federated STDP framework on distributed nodes to evaluate synchronization overhead, scalability, and robustness under non-IID data conditions.

This roadmap provides a clear path toward experimental validation, ensuring that future studies will comprehensively benchmark the proposed architecture under realistic and scalable conditions. Quantitative comparison of neuromorphic hardware and ANN systems is tabulated in Table 6.

**Table 6:** Quantitative comparison of neuromorphic Hardware and ANN systems.

**Platform**	**Scale & Arch**.	**Neurons**	**Proc**.	**Energy / Event**	**Latency / throughput**	**On-chip**	**Power**	**Application**
Intel Loihi 2 Davies et al., 2021	Neuromorphic SoC	~1M	4 nm	100 × lower vs CPU	< 1 ms	Yes	23.6 mW	Sensor fusion; LLM inference
Loihi Hala Point Uludağ et al., 2024	1152-chip cluster	~1.15B	4 nm	100 × lower vs GPU	50 × faster	Yes	2.3 kW	Datacenter neuromorphic compute
BrainChip Akida Lutes et al., 2025	Edge SoC	~1.2M	28 nm	sub-μ J/inference	real-time	No	1 W	Edge AI; cybersecurity
SpiNNaker Yan et al., 2019; Furber and et al., 2014	ARM mesh cluster	~1M/board	130 nm	8 nJ/event	1:1 wall-clock	Yes	80 kW	Robotics; neuroscience sim
Loihi (U-Net) Roy et al., 2019	SNN U-Net	~10M	14 nm	2 × more efficient	comparable	Yes	–	Image segmentation
Loihi 2 (LLM) Schuman et al., 2022	Transformer SNN	~1M	4 nm	2 × less energy	3 × faster	Yes	–	LLM inference
GPU/CPU ANN Mead, 1990	Dense inference	N/A	5–7 nm	10–100 mJ/inference	10–50 ms	No	100–300 W	Vision; NLP; HPC

Finally, we emphasize that these results are intended as illustrative. In our proof-of-concept experiments with the NSL-KDD dataset, the SNN achieved lower accuracy than conventional ML baselines. This outcome is consistent with the current state of neuromorphic research, where the primary advantages of SNNs lie in event-driven efficiency, low latency, and energy savings rather than maximizing classification accuracy in early prototypes. The presented results should therefore be viewed as evidence of feasibility within the proposed Digital Twin framework, with future work focusing on enhanced encoding strategies, larger-scale networks, and hybrid neuromorphic–ML approaches to improve accuracy while retaining the neuromorphic benefits.

## 9 Comparative analysis with traditional security models

To contextualize the performance and novelty of the Neuromorphic Cyber-Twin, Table 7 presents a comparative analysis against conventional artificial neural network and machine learning based security models commonly applied in Digital Twin environments. This comparison highlights the advantages of NCT in latency-critical, resource-constrained, and dynamically evolving cyber-physical system (CPS) scenarios. Traditional approaches often rely on centralized training, large labeled datasets, and periodic retraining to adapt to new threats. By contrast, the NCT employs spiking neural networks (SNNs) with spike-timing dependent plasticity (STDP) to achieve event-driven, on-device adaptation. This enables decentralized decision-making, rapid response to emerging anomalies, and significantly reduced energy consumption.

**Table 7:** Comparison of neuromorphic Cyber-Twin with traditional DT security architectures.

**Feature**	**ML-based DT security**	**ANN-based IDS**	**Neuromorphic Cyber-Twin (NCT)**
Learning mode	Supervised	Offline batch learning	Unsupervised online learning
Latency	Medium	High	Ultra low (event-driven)
Energy efficiency	Low (cloud-dependent)	Medium (GPU-intensive)	High (event-driven neuromorphic)
Adaptivity	Weak (requires retraining)	Moderate (partial re-training)	Strong (via STDP-based plasticity)
Hardware Fit	Cloud / Edge GPU	Cloud CPU / GPU	Edge Neuromorphic SoC (e.g., Loihi, Dynap-SE)
Explainability	Moderate (feature importance)	Low (black-box models)	Moderate (traceable spike activity)
Context awareness	Weak (data-driven only)	Weak (static features)	Embedded (via feedback-memory loop)

Adversarial Robustness: While these benefits underscore the potential of the NCT, adversarial robustness remains a critical open challenge. Neuromorphic systems may still be susceptible to evasion attacks, where carefully crafted spike patterns mimic benign telemetry, and poisoning attacks, where malicious updates compromise federated STDP aggregation. Promising countermeasures include adversarial training with perturbed spike encodings, anomaly-aware STDP rules resistant to gradient-based manipulation, and trust-weighted aggregation strategies in federated learning. Addressing these aspects is an essential direction for strengthening the resilience of NCT deployments.

## 10 Conclusion and future work

Digital Twins are becoming indispensable in modern cyber-physical systems, enabling real-time modeling, monitoring, and autonomous control. Yet, as these systems scale in complexity and interconnectivity, they are increasingly exposed to dynamic cyber threats that traditional static security models cannot address. This paper introduced the Neuromorphic Cyber-Twin, a brain-inspired architectural framework that embeds cognitive security mechanisms directly into DT environments. By leveraging spiking neural networks, event-driven computation, and biologically plausible learning rules such as spike-timing-dependent plasticity (STDP), the NCT enables low-latency, adaptive, and energy-efficient defense strategies capable of real-time anomaly detection and autonomous response.

The NCT architecture has been positioned across a range of critical use cases, from smart grids and vehicle-to-everything (V2X) systems to medical cyber-physical devices and federated smart city infrastructures. Compared to conventional machine learning-based detection methods, the NCT demonstrates unique advantages in its unsupervised learning capabilities, contextual adaptability, and deployment feasibility on edge neuromorphic hardware.

Looking ahead, several directions offer promising opportunities for advancing this work. These include the development of functional prototypes on neuromorphic platforms such as Intel Loihi and BrainChip Akida, and the simulation of spiking models using frameworks like Brian2. Another critical avenue involves the generation of synthetic DT telemetry datasets with embedded anomalies to enable benchmarking and validation of neuromorphic security mechanisms. The extension of the NCT to support federated learning architectures will also be essential, enabling secure, distributed adaptation across decentralized DT ecosystems. Moreover, hardware–software co-design approaches are needed to embed SNN inference into low-power edge platforms while maintaining real-time responsiveness. Finally, the creation of unified toolchains that integrate Digital Twin simulation environments with neuromorphic processing backends will be vital for streamlining development and deployment.

In summary, the NCT lays the foundation for a new class of intelligent, self-defending digital systems. By fusing neuromorphic computing with dynamic cyber-physical infrastructures, it opens a compelling direction for the future of resilient, context-aware security in virtualized ecosystems.

## Data Availability

The original contributions presented in the study are publicly available. All code, sample telemetry, and simulation outputs used in this study are publicly available at https://github.com/DrNidaNasir/Neuromorphic-Cyber-Twin-NCT-.
